# Friction Determination by Atomic Force Microscopy in Field of Biochemical Science

**DOI:** 10.3390/mi9070313

**Published:** 2018-06-21

**Authors:** Yan Wang, Jianhua Wang

**Affiliations:** College of Bioengineering, Chongqing University, Chongqing 400044, China; 20161901026@cqu.edu.cn

**Keywords:** atomic force microscopy (AFM), friction, biochemical science

## Abstract

Atomic force microscopy (AFM) is an analytical nanotechnology in friction determination between microscale and nanoscale surfaces. AFM has advantages in mechanical measurement, including high sensitivity, resolution, accuracy, and simplicity of operation. This paper will introduce the principles of mechanical measurement by using AFM and reviewing the progress of AFM methods in determining frictions in the field of biochemical science over the past decade. While three friction measurement assays—friction morphology, friction curve and friction process in experimental cases—are mainly introduced, important advances of technology, facilitating future development of AFM are also discussed. In addition to the principles and advances, the authors also give an overview of the shortcomings and restrictions of current AFM methods, and propose potential directions of AFM techniques by combining it with other well-established characterization techniques. AFM methods are expected to see an increase in development and attract wide attention in scientific research.

## 1. Introduction

Atomic force microscopy(AFM) is an analytical technology based on the interaction between surfaces and the tip at a single molecule level. The scanning tunneling microscope (STM) was developed in 1982 by Binning and Rohrer to take images of surfaces at the atomic level. However, STM requires samples to have electrical conductivity for measurement. To overcome this disadvantage, Binning and co-workers subsequently developed AFM in 1986. AFM uses a micro cantilever as the transmission medium of force signals. The AFM consists of a piezoelectric element, probe tip, detector, feedback system and control system [1]. It can be used to image local surface characteristics and evaluate dynamic properties from microscale to nanoscale. Compared to other measurement technologies, AFM offers high selectivity, accuracy, and resolution up to the atomic level [2,3,4]. It is a non-destructive analytical technique and can be used in various environments without sample-processing as air, liquid, and vacuum [5,6,7]. AFM can be widely applied for the measurement of mechanical, electrical and magnetic properties of chemicals at the nanoscale [8,9,10]. In addition, the functional software of AFM systems also provide quantitative information in characterization of mechanic properties [11,12,13,14]. Therefore, AFM has been widely applied for measurement of sample morphology and atomic forces, especially longitudinal forces. While friction force and frictional properties of samples were previously measured by other methods, AFM was commonly used to characterize thickness, morphology [15,16,17], roughness [15,18,19,20] and surface characterization. As a useful tool, AFM is increasingly being used to measure the interaction forces between samples and recognition events between proteins at the single molecule level. It is now also being used for measurement of interactions between antigen and antibody [21,22], misfolding of protein [23,24,25], and protein-DNA interactions [26,27,28].

However, there is little application for lateral force measurements (such as friction force) [29,30,31,32]; thus, there is not much comprehensive systematic review on friction determination by AFM. This article is based on the measurement of friction by AFM at the single molecule level in the field of biochemical science. This paper has summarized recent researches and processes of the AFM method in friction determination, embodying its application in the biochemical field. The paper also discusses some problems of AFM and prospects of research in this field, offering certain recommendations and advice for further research.

## 2. Mechanism of Friction Mechanics and Measurement Principle at Nanoscale by Using AFM

From the early Amonton’s Law to medium-term Coulomb’s friction law, friction mechanism has been developed through a long process with perfection of mechanical meshing theory and furrow effect. However, friction theory is not applicable at the nanoscale due to the surface effect, size effect, and quantum effect. The Tomlinson’s model of molecule action theory has gradually become the basis of friction mechanism at the nanoscale. Several new model applications based on Tomlinson’s model have emerged, which include Thermal Tomlinson’s model, Frenkel-Kontorova’s model, and Frenkel-Kontorova Tomlinson’s model. Nano-tribology is mainly based on molecular and atomic structure. It is considered by friction behavior on the nanoscale surface and interface molecular layer. The research foundation is surface physics and surface chemistry. For contemporary experiments, the friction measurement at nanoscale is based on the development of the Tomlinson’s model [33,34,35,36]. Since the late 80s, AFM (FFM) as a new experimental technique has been widely applied to the determination of friction at nanoscale [37,38,39,40,41].

Friction properties are determined by the mechanical properties of soft materials, which makes it easy to measure the friction between probe and samples. Taking into consideration the flexibility and softness, friction between probe and samples is determined by the mechanical properties of the sample surfaces [42,43,44]. The friction properties of sample surfaces can be obtained via the measurements.

**Figure 1:**
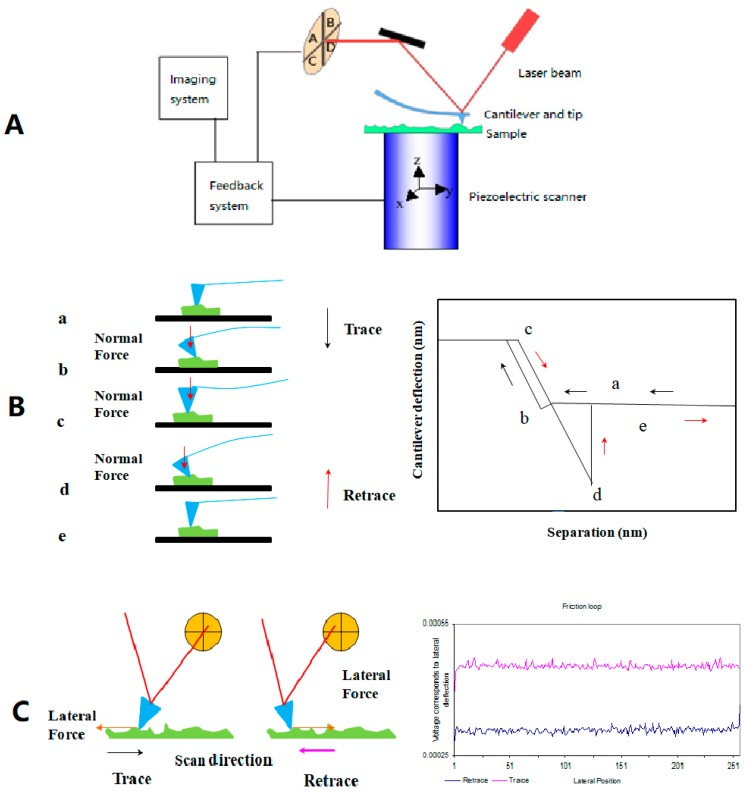
Schematic diagram of force measurement by AFM: (**A**) Structure diagram of AFM, including laser emitting and receiving system, piezoelectric scanning system (cantilever and tip included), imaging system and feedback system; (**B**) The principle of measuring normal force and the typical force-distance curve; (**C**) The principle of measuring lateral forces and the typical, friction-loop curve.

For the friction mode of AFM, some reports directly describe it as lateral force microscopy (LFM) or friction force microscopy (FFM). Due to the presence of lateral force between the sample surface and sharp diamond tip mounted on a stiff cantilever beam, the cantilever has a lateral torsion under force [45]. The laser beam deflection caused by the cantilever is measured by a quadrant photodiode of the optical system. The normal load and the friction forces are respectively proportional to the normal and torsional deflections of the cantilever, recorded simultaneously via the output of the photodiode detector [46]. As shown in Figure 1, the signal voltage of (A + B) − (C + D) and (A + C) − (B + D) reflects the vertical bending and horizontal torsion, respectively. Normal and lateral forces can be calculated with known parameters and formula by converting electrical signals to mechanical signals.

## 3. Friction Measurement by AFM

It has been demonstrated that the research process of frictional determination by AFM in the biochemical field in the past decade was mainly focused on biochemical materials at the nanoscale.

AFM has proved to be a valuable tool in the investigation of the surface structure of the human hair and is routinely used to assess the effectiveness of cosmetic treatments [47,48,49,50,51,52]. In addition, AFM is widely used in the study of the tribological properties of biological tissues, identification, and screening of biomaterials. The advantages of AFM technology in the determination of biological samples mainly include these aspects, but are not limited to simplification, relative physiological condition and miniscule damage. AFM has undergone an evolution from determination of frictional morphology to various mechanical parameters of friction value and process. The two aspects that determine dynamic and static properties can be achieved by multi-AFM modes. Here we specify the progress of three measurements at nanoscale using experimental cases: frictional morphology, friction curve and friction motion process.

### 3.1. Frictional Morphology

When the influence of a solution environment on biological samples (such as protein) is measured, morphology deservedly becomes the most typical measurement. However, in a solution environment, it is difficult to observe changes in morphology and structure in extremely fine sizes, when differences in friction morphology are obvious. Friction morphology holds clarity, brightness and precision at a high level in a visual field.

**Figure 2:**
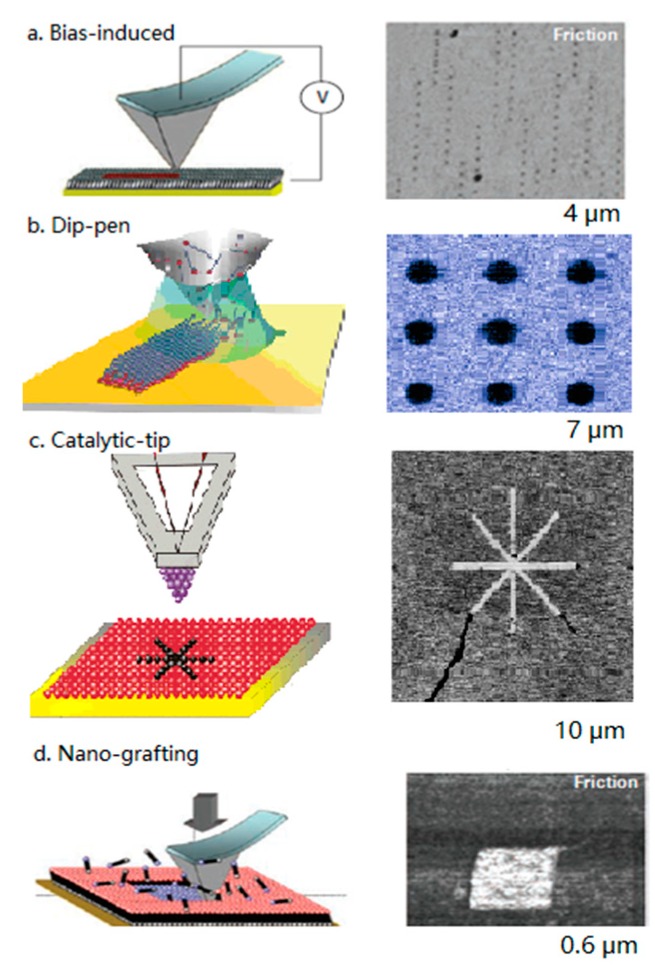
Comparison of SPL methods with friction morphology for SAM: (**a**) In bias-induced nanolithography; (**b**) in dip-pen nanolithography; (**c**) in catalytic-probe lithography; and (**d**) in nanografting pattern. (**a**–**d**) Adapted respectively with permission from [54,55,56,57,58].

Jayne C. Garno [53] and co-workers developed the scanning probe lithography (SPL), offering new possibilities for nanoscale investigations of protein binding. They summarized the progress of AFM method with nanofabrication of functionalized tips. Based on scanning probe lithography, it was expected to develop nanoscale protein assays to attain the ultimate miniaturization by using AFM. In addition to their emphasis that SPL enables researchers to engineer spatial parameters with nanometer precision to place molecules of well-defined composition, it is convincible now that friction can be visualized with maximum resolution and precision. Figure 2 shows a summary of friction morphology measured by AFM with different scanning probe lithography [54,55,56,57,58]. On completing tip modification with various SPL techniques, AFM was utilized for morphology and friction imaging. All measurements were in an air environment with scan size at microscale. Friction morphology of self-assembled monolayer (SAM) reveals great performance of easy and clear visualization, while surface images are not comparable.

**Figure 3:**
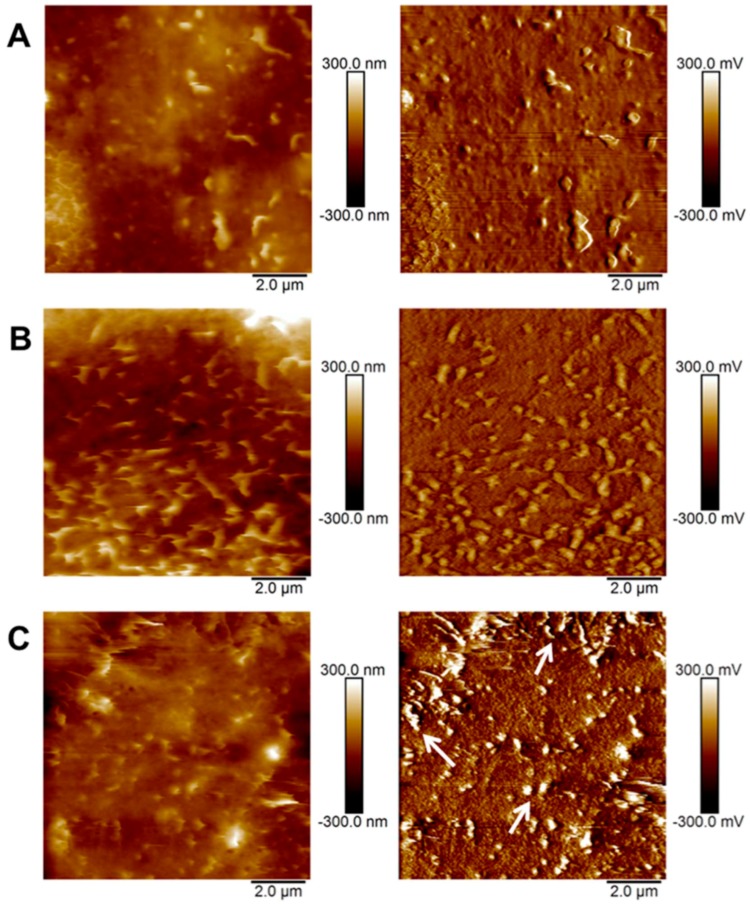
AFM topography (left) and friction (right) images of the in vitro model (TR146/MUC1 cells with a mucosal pellicle). (**A**) Control (no EgCG); (**B**) EgCG 1 M; (**C**) EgCG 3 mM. Adapted with permission from [59].

As shown in Figure 3, Ployon and co-workers presented [59] AFM images and friction forces of the oral mucosa model’s surface in control condition (no tannin) and in the presence of a dietary tannin EgCG at 1 and 3 mM. The topography and friction images were collected in phosphate buffer saline (PBS) medium with V-shape silicon nitride cantilevers in contact mode with high resolute (512 × 512 pixel^2^). The scan rate was 1 Hz and scan size was 10 × 10 μm^2^. The topography of the in vitro model exposed to 1 or 3 mM EgCG was not drastically modified compared to the control condition (left). Fiction force (right) appeared influenced by EgCG concentration. At 3 mM especially, some areas were characterized by higher friction force (see arrows) than the other conditions. By contrast, changes of protein were reflected through friction morphology in the highlighted sections while morphology (left) did not show visual clarity.

Measurement of friction morphology was also mentioned in a paper previously published [60]. The adaptability and operability of friction imaging using AFM method in a liquid environment make AFM a favorable tool in studying morphology, structure, and conformation of biochemical samples.

### 3.2. Friction Curve

In majority of experiments on determination of frictional properties, friction force value and friction coefficient were obtained by AFM. In typical research, frictional characteristics can be analyzed by friction curve versus load friction. In most experimental cases, the friction properties of sample surfaces were analyzed through friction curve versus load force. Friction force was proportional to the load force, which basically complied with classical friction law. The friction curve versus load force invariably fitted the positive function when changing contact interface, scanning velocities, environmental condition, or probe specification in the listed examples. All measurements presented in the following paragraph were performed in an air environment (except for special demonstration) with high resolute (512 × 512 pixel^2^). Though the AFM instruments varied among the experimental cases, when using a bare probe in determination, the V-shaped Si_3_N_4_ cantilevers (nominal spring constant of 0.06 N/m) and square pyramidal tips with an end-tip of 30–50 nm radius used were constant, except for the last two cases, wherein the rectangular cantilever was used. In the last two experimental cases, the cantilever was nanofabricated with sphere, and to avoid damage to the surface, the rectangular shape cantilever was chosen, due to its low spring constant. Friction calculations were based on JKP (Johnson, Kendall and Roberts) theory.

By measuring the friction properties of virgin, damaged and conditioner-treated hair at three scales, LaTorre and coworkers [61] investigated the scale effects and directionality dependence of friction. All measurements were performed using a commercial AFM system (MultiMode Nanoscope IIIa, Digital Instruments, Santa Barbara, CA, USA) in ambient conditions. They have explained in details about the effect mechanism of conditioner at different scales of hair. Similarly, Smith [62] observed friction of human hair cuticles by using a Discoverer TMX 2000 scanning probe microscope. The authors have analyzed the relationship between friction forces and structure of cuticles, and clarified that the friction values varied in three layers because of the differences of softness. Both studies provided a theoretical basis for the selection of washing products. Force measurements were presented after force calibrating, based on methods previously published [63,64].

Some materials have increasing application in multifarious study owing to biologic properties like non-toxicity, biocompatibility and absorbability. Mcnamee and co-workers [65] compared the friction forces between grafted polysaccharide layers, both in the absence and presence of surfactants. Image and friction force were measured by Nanoscope III Multimode miscroscope separately in the tapping and friction mode. The results showed that friction decreased with lubrication effect and electronic effect. The author concluded that emulsifiers can affect the friction properties of biological materials by reducing the thickness of the biologic film with lubrication, chemical binding and electronic effect.

**Figure 4:**
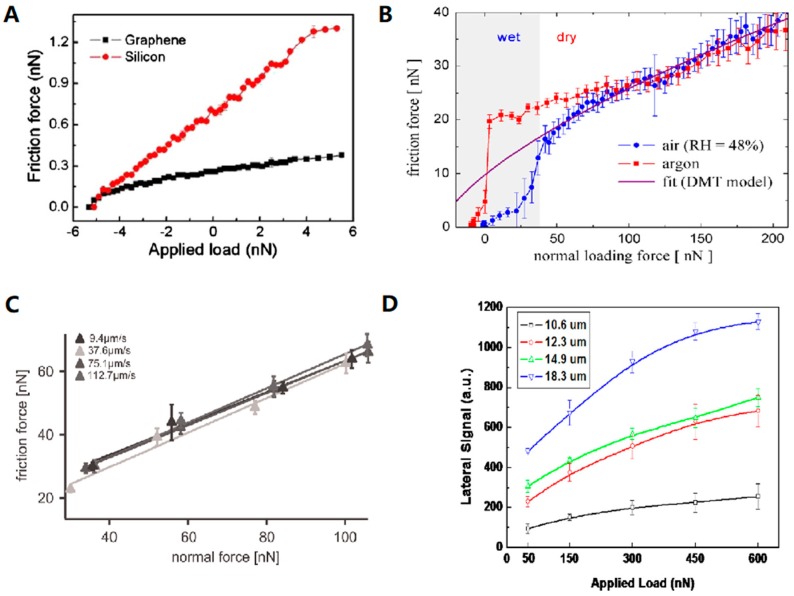
Friction curve versus applied normal load force under different condition: (**A**) at different material surfaces [66]; (**B**) in different measurement environments [70]; (**C**) at different scanning velocities [71]; (**D**) at flat surface measured with colloidal probes of different lengths [72]. (**A**–**D**) Adapted with permission from [66,67,68,69,70,71,72], respectively.

Since grapheme has been widely applied in nanotechnology, it is of interest to thoroughly understand its nanoscale tribological characteristics as well. Lin and co-worker*s* [66] investigated friction characteristics of multi-layer grapheme films by AFM in contact mode. The frictional properties were obtained from applied load cycles from negative to balance and then to positive, applied between AFM tip/specimen contacts. Grapheme films exhibited much lower friction than bare Si surface. Figure 4A shows an example of the friction force versus applied load of two different substrates. The authors indicated that grapheme was an attractive material for its perfect frictional properties. The low-frictional property of graphite was attributed to the formation of a thin layer and weak bonding between the basal planes, which led to its lubricious property [67]. Besides, friction characteristics of graphite are strongly influenced by the environment and water vapor was required for passivation of graphite surface to maintain low friction [68,69].

The friction between hydrogen-free diamond-like carbon thin film surfaces was observed by Sirghi [70]. As shown in Figure 4B, the friction force versus normal loading force indicated that the former was much lower in humid air when the relative humidity (RH) was 48% higher than in argon environment. They contributed this effect to the formation of a water layer absorbed on the film surface in humid air. The water layer played a lubricating role in lowering friction on the film surfaces.

Kienle [71] studied the effects of different lubrication types on the friction and wear of articular cartilage. Before friction measurement, cantilever was nanofabricated with a polystyrene sphere attached. Measurements were performed in a liquid environment by using AFM (MFP-3D SA, Asylum Research, Santa Barbara, CA, USA) in contact mode. The linear relationship between the friction force and the normal force at different scan velocities was observed (Figure 4C). Although it has been recognized that available viscosupplements are useful in managing osteoarthritis (injection of hyaluronic acid reduces pain and friction), hyaluronic acid does not significantly lower friction while improving wear resistance.

Similarly, frictional properties on different film surfaces were measured with modified colloidal probe by AFM (AIST-NT SmartSPM, Novato, CA, USA) in Lateral Force Microscope (LFM) mode [72]. The results indicated that friction for silicon dioxide nano-textured polyimide film was lower than flat polyimide film (Figure 4D). The contact area between the sample surface and probe was considered as the main reason. The authors evaluated the effect of colloidal probe size on friction behavior and further observed that friction increased with increase in probe size, further confirming that the contact area affected friction value.

Multifunctional AFM has been widely applied in determination at the nanoscale. Friction curve, as the most representative measurement for friction, is demonstrated in many previous papers. There are only a few research reports that focused on the measurement of friction motion process, which is perhaps more valuable in measuring tribological properties of nano-materials.

### 3.3. Friction Motion Process

**Figure 5:**
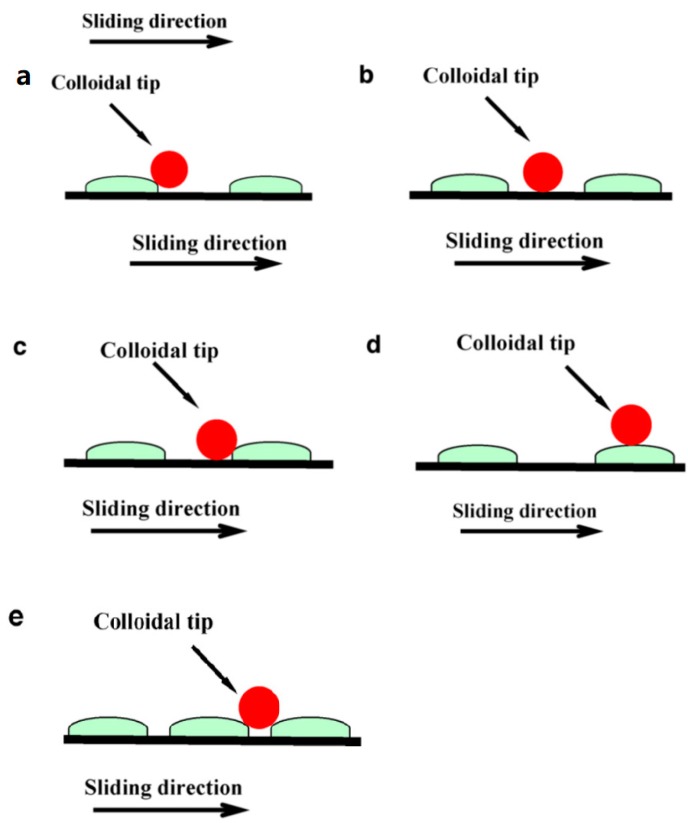
Schematic diagram of the contact between the colloidal probe and patterned surfaces: (**a**) point-point contact; (**b**) point-plane contact; (**c**) multi-contact; (**d**) point-point contact; (**e**) multi-contact. Adapted with permission from [73].

Considering the generality of friction curve, the determination of the friction process is still emerging as the times require for certain reasons: analysis of the mechanism, complete evaluation of friction properties, and consideration of the influence on friction of the contact between probe and sample surface. Here shown in Figure 5, we use a published study of tribological behavior of micro/nano-patterned surfaces in contact with AFM colloidal probe as an example [73]. Contact diversity in determination is universally existent in studies, which influences friction force value during determination. Under such a circumstance, it is not comprehensive to evaluate tribological characteristics merely by measuring friction morphology or friction value.

**Figure 6:**
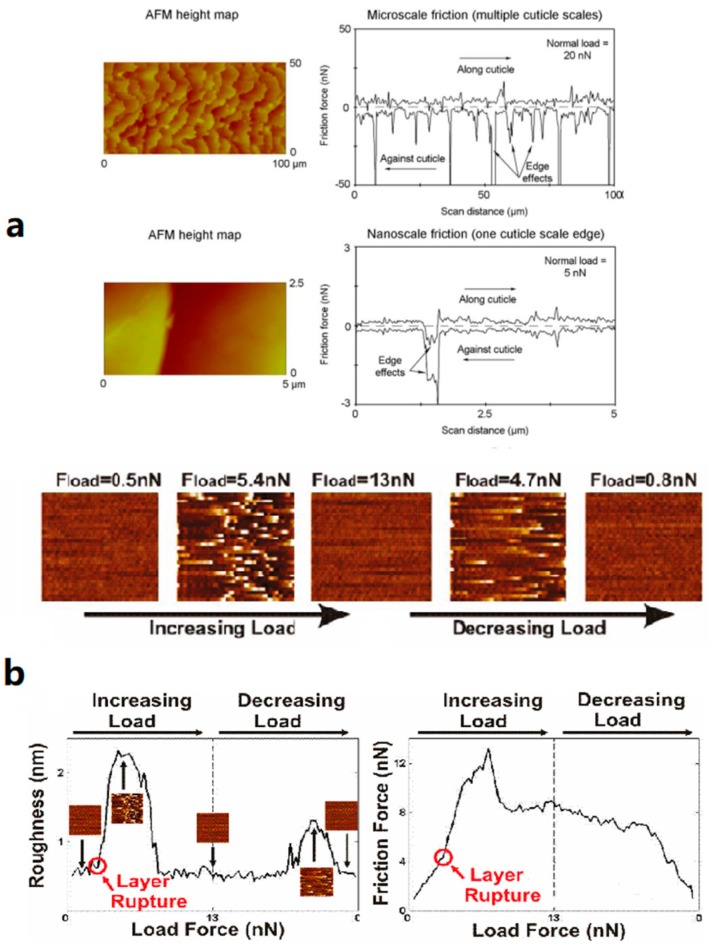
Friction motion process of biological samples in different orders: (**a**) Directionality effects of hair friction at microscale (top) and nanoscale (bottom) reported through friction loop during the scanning process. Adapted with permission from [61]. (**b**) Simultaneous determination of topography and friction force (right) or roughness (left) and versus load force of the scanning process. Adapted with permission from [74].

Figure 6 shows the friction motion process of two different kinds of biological samples: the macroscopical biological sample of human hair and the microscopical biological sample of proteins. Figure 6a was adapted from an investigation by Latorre and coworkers [61] on scale effects and directionality dependence on friction of human hair using AFM to measure the changes of friction force through a dynamic process. Since there were both similarities and differences when comparing the tribological trends at both scales, the scale effect was considered as an important aspect of studying the tribology of hair to bridge the gap between the macroscale and nanoscale data, and to discuss the mechanisms behind the scale effects.

Figure 6b was adapted from a study on the strength of biological surfaces through simultaneously monitoring of topography and friction between probe and protein samples. Stores and co-workers [74] implemented friction force microscopy as a tool to measure the dynamic friction characteristics. A commercial AFM equipped with a liquid cell was employed (Multimode SPM with a Nanoscope IV control unit, Veeco Instruments, Santa Barbara, CA, USA). The measurement provided an idea that can effectively restore the relationship between friction and morphology. The strength and lateral diffusion of a protein surface has been proved to have a certain relationship to pH, as indicated in the paper.

Khan and co-workers monitored in real time the fusion of large unilamellar vesicles and lateral organization of lipid molecules by using AFM before and after the formation of supported lipid bilayers (SLBs). Herein, SLBs were used to probe the functionality of biological membranes. Furthermore, they estimated bilayer thickness and calculated the rupture force at the interface of the tip and the SLBs using the cantilever tip in AFM. They ultimately anticipated that a silicon-based micron-sized cavity had the potential to maintain the stability of well-formed membranes inside [75]. Moreover, they used AFM to characterize the presence and thickness of supported lipid membrane (SLM) on the nanopore chip in another functionality measurement of SLM. It follows that AFM can be a useful tool for functionality characterization of biological membranes [76].

In addition to biological samples, chemical samples including macroscopical and microscopical materials were similarly measured by friction process. Titania surface and nanowire are used as two typical examples, respectively, to reveal size specification from works previously published.

**Figure 7:**
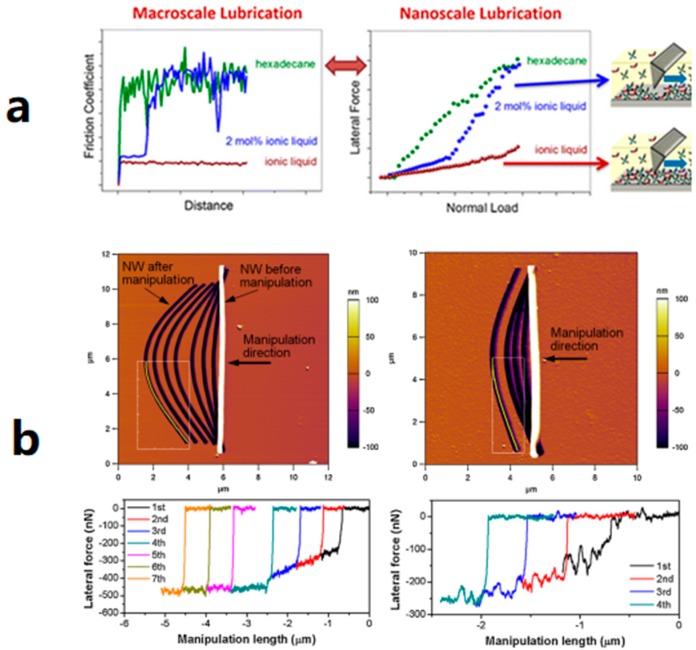
Friction motion process of chemical samples on different orders: (**a**) Dynamic variation of friction coefficient against distance at macroscale (left) and lateral force against normal load at nanoscale (right). Adapted with permission from [77]. (**b**) Subtracted AFM images of oxidized Si NWs on SiO_2_ substrate (left) and graphene substrate (right) along with variations in lateral forces during manipulations. Adapted with permission from [78].

Li and co-workers [77] compared the lubrication of the titania surface by pure hexadecane, pure ionic liquid (IL), and a mixture of IL and hexadecane. Nanotribology measurements were performed using a Bruker NanoScope VIII Multimode AFM. Three sharp silicon cantilevers (spring constant = 2.0 ± 0.2 N/m by the thermal tune method, tip radius ~8 nm) were used in lateral force measurements in contact mode at a scan angle of 90°. The authors found that IL was a more effective lubricant to decrease the friction of the titania surface at both macroscale and nanoscale. The significant lubricating effect of IL on the titania surface was observed (Figure 7a). The lubrication performance of the hexadecane was slightly improved by adding a small dose of IL. They, therefore, recommended the industrial application of IL lubricant for titania and other alloys. In this measurement, they reduced the dynamic friction motion by increasing loading force. Optimum selection of lubrication can be perorated from the dynamic friction curve in nanotechnology.

The role of graphite as a lubricant was also mentioned in a study on static and kinetic friction properties of nanowires (NWs) on different substrates [78]. Kim measured and compared the static and kinetic friction of oxidized Si NWs deposited on SiO_2_ and grapheme layers, respectively, using a commercial AFM (MFP-3D, Asylum Research, Santa Barbara, CA, USA). The Si AFM cantilevers with a normal spring constant of 9 N/m were used under constant force mode to obtain friction force during the manipulations. The substrate AFM images with variations in lateral forces during manipulations are accurately displayed in Figure 7b. This finding enhanced the understanding of friction properties of NWs and friction at nanoscale, and therefore, helped improve designs at nanoscale of nano-devices from a tribological point of view.

### 3.4. Other AFM Measurements on Friction

In addition to the three measurements aforementioned—friction morphology, friction curve and friction motion process—AFM was also applied in measuring friction characteristics with other measurements in experimental cases.

The influence of ionic adsorbents on friction forces at single crystal electrodes was studied by Hausen [79]. It is observed that friction was positive to potential when absorbed Cu increased coverage on Au crystal electrodes. The research opened a novel method for studying ionic adsorbents by considering the mobility, stiffness and effect on surface tension. Similarly, a study on anisotropy effects on the friction force at a regular step Au electrode using AFM was reported [80]. With increasing Cu coverage, the friction value increased when the tip was scanning parallel but not perpendicular. Both measurements were performed with a commercially available Nanoscope III E controller (Digital Instruments, Santa Barbara, CA, USA) fitted with an electrochemical cell. The nominal spring constant of the commercial Si cantilevers was 0.9 N/m.

Liu compared the friction properties on graphite surfaces using two different methods [81]: theoretical simulation and experimental determination. Experimental friction was measured with a commercial AFM system (Multimode, Bruker, Santa Barbara, CA, USA) and rectangular cantilevers with normal spring constant of 0.2 N/m. The resolution of image was kept constant of 256 × 256 pixel^2^. In simulation, one atom was used to represent the tip, and spots obtained in the spectrum revealed the main wave components in friction force patterns. The relationship between the friction and length in both situations of experiment and stimulation were constant. The agreement of the results demonstrated that friction force pattern could be interpreted reliably with models. The paper also questioned crystal material properties reported in previous studies, i.e., thermal drift in the experimental process. Due to the thermal drift, the offset angle between AFM motion coordinate and crystal lattice change could not be quantitatively determined in the experiments.

Jabbarzadeh [82] conducted experiments with SAMs of n-alkanethiols to investigate friction anisotropy and asymmetry on film surfaces, and verified the existence of friction anisotropy and asymmetry. This finding was completely in agreement with the stimulation. Therefore, it was concluded that friction was dependent on the direction of sliding and molecules tilt, which exhaustively explained the mechanism of the phenomenon.

## 4. Conclusions

This article summarizes the progress of AFM over the past decade as a measurement tool for frictional characteristics in the field of biochemical science. AFM has wide applications by virtue of properties such as high precision, high resolution, non-destructivity, and functionality. In the study of friction mechanics, AFM performs universally in surface characterization and force determination. It has played an important role in evaluating surface properties, especially for nanomaterials. It is expected to be a useful tool for identification and screening of materials. However, there are several limitations. The application area is limited mainly to the field of material science and mechanics. Because of the requirement for a special experimental environment, time and resolution, the application of AFM in the field of biology is not of wide prevalence. AFM may be used to study the effect of force on cells, the mechanical properties of macromolecules such as proteins, and DNA, which may be the basis for studying the mechanical properties of tissues and organs. AFM can be an important tool for the study of human diseases, health, and the exploration of the development of life science. In order to broaden its applications, it is necessary to combine AFM technology with other well-established characterization technologies. For example, AFM may be used to measure biochemical reaction in vivo (when combined with high-speed and dynamic technologies); or for characterization of more complex systems (when combined with Roman, TEM, NMR, SEM and other characterization methods). In addition, the accuracy, precision and resolution of AFM can also be indispensable factors that influence research results. Therefore, eliminating unnecessary errors and improving the resolution of images is an urgent problem that needs to be solved. It is reasonable to believe that if these difficulties are overcome, the AFM method could be utilized in various analyses and determination processes.
